# Supplementary figures and images for: A local measure of symmetry and orientation for individual spikes of grid cells
Source: PLoS Comput Biol. 2019 Feb 7;15(2):e1006804. doi: 10.1371/journal.pcbi.1006804 (PMC6382163; doi:10.1371/journal.pcbi.1006804)

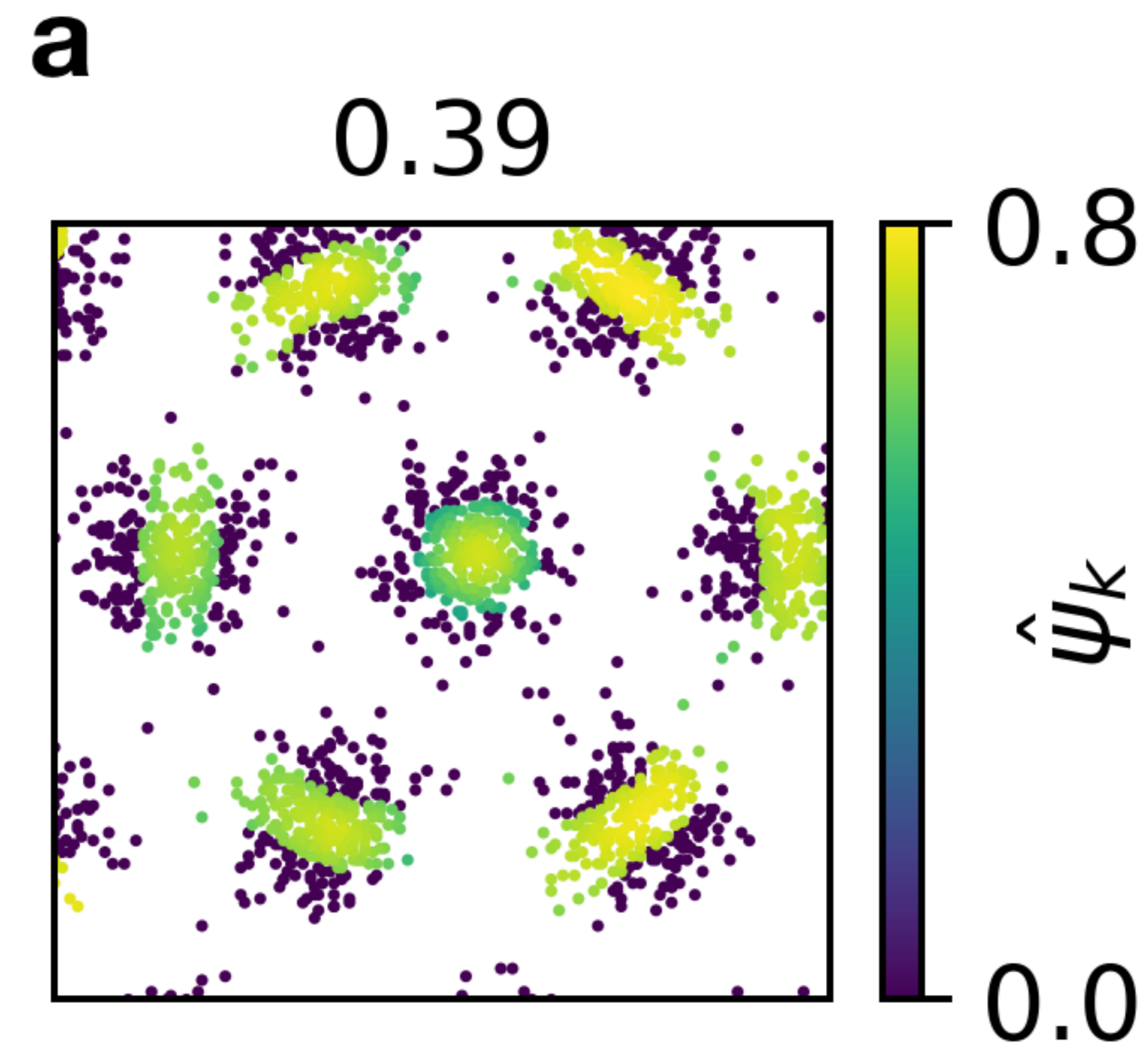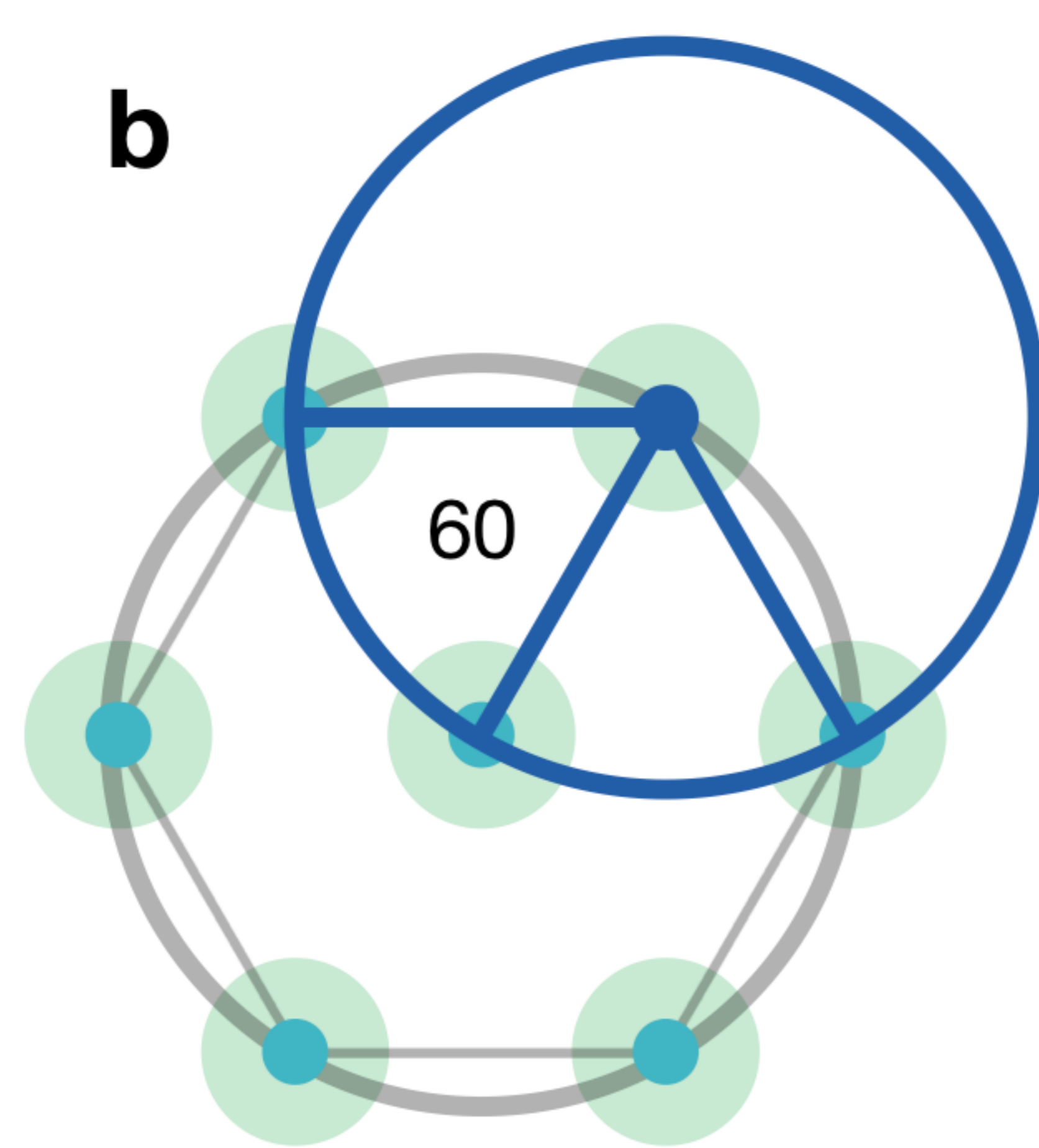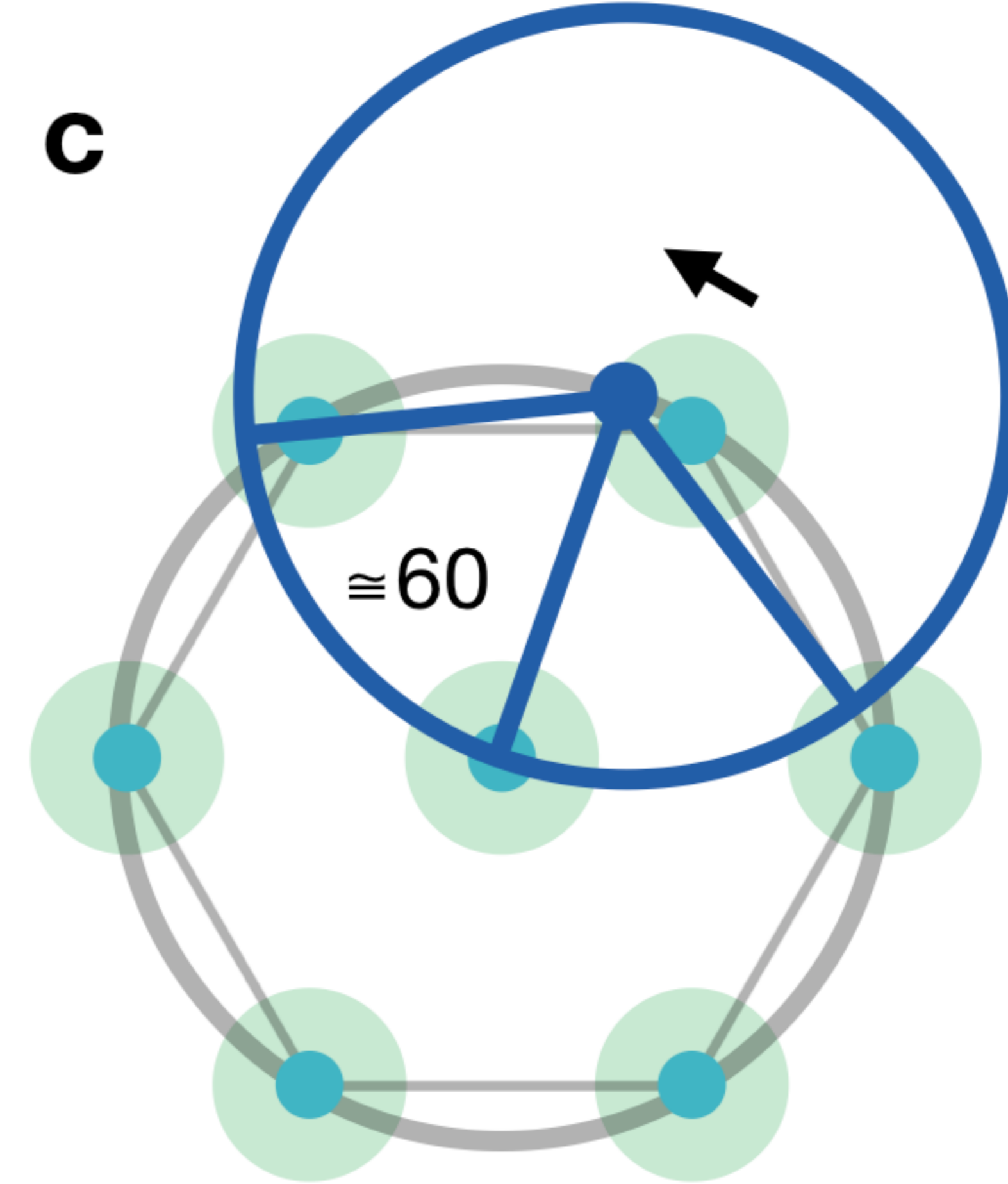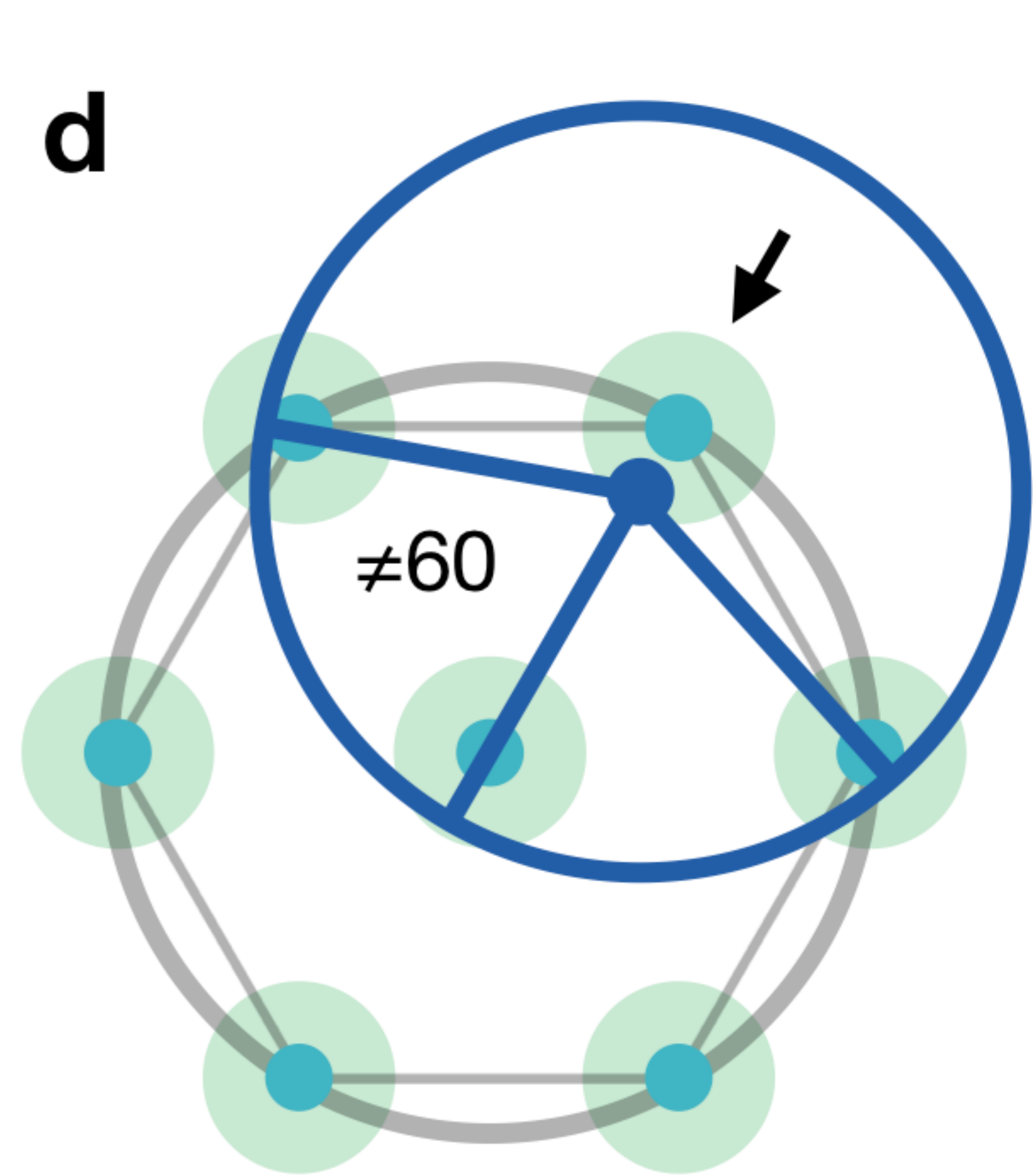

Supplement: S1 Fig — a) Generated spike locations with few firing fields. The color of each spike k indicates the ψ^k score. Fields at the boundaries have only three neighboring fields. This leads to a stripe-like assignment of ψ^k scores. b) Schematic of a perfect grid cell. Dots on the hexagon mark the centers of grid fields. Shaded regions show the grid field size. The dark blue dot in the upper right marks a reference spike in the center of a grid field. The dark blue circle around it indicates the neighborhood shell—much thinner than actual, for easier visualization. Connecting the reference spike to the center of the cross section of the neighborhood shell and the neighboring grid fields forms angles of 60 degrees. c) Same arrangement as in b but now the reference spike is not in the center of the grid field, but shifted along the gray circle that circumscribes the hexagon (direction indicated by arrow). Connecting the reference spike to the center of the cross section of the neighborhood shell and the neighboring grid fields still forms angles of roughly 60 degrees. Consequently, spikes along this direction have high ψ^k scores. d) Same arrangement as in b, c but now the reference spike is shifted orthogonally to the circle that circumscribes the hexagon. Connecting the reference spike to the center of the cross section of the neighborhood shell and the neighboring grid fields does not form angles of 60 degrees. Consequently, spikes along this direction have low ψ^k scores. For central grid fields, this effect is compensated by grid fields on opposite sides. (PDF) [file pcbi.1006804.s001.pdf]

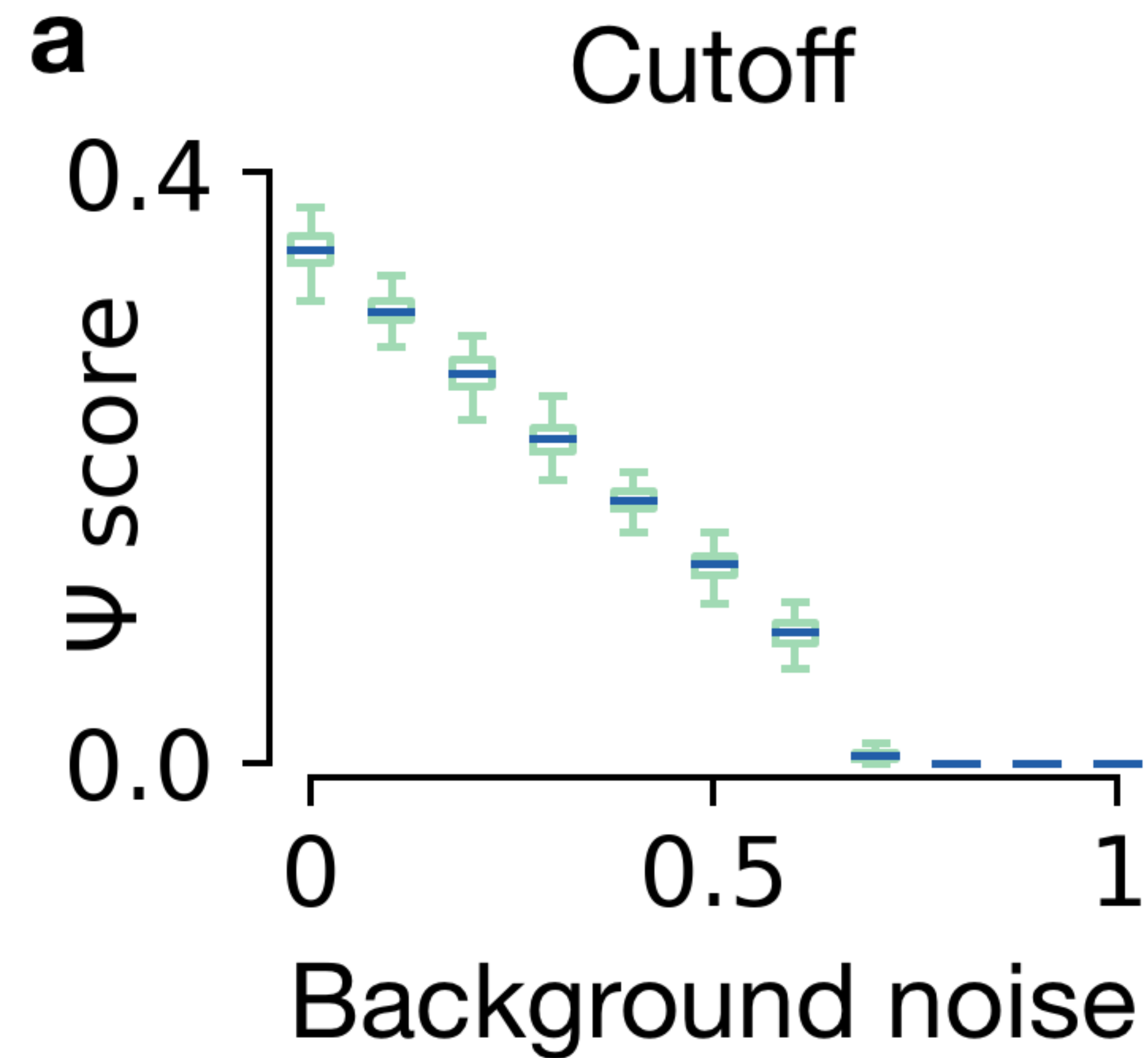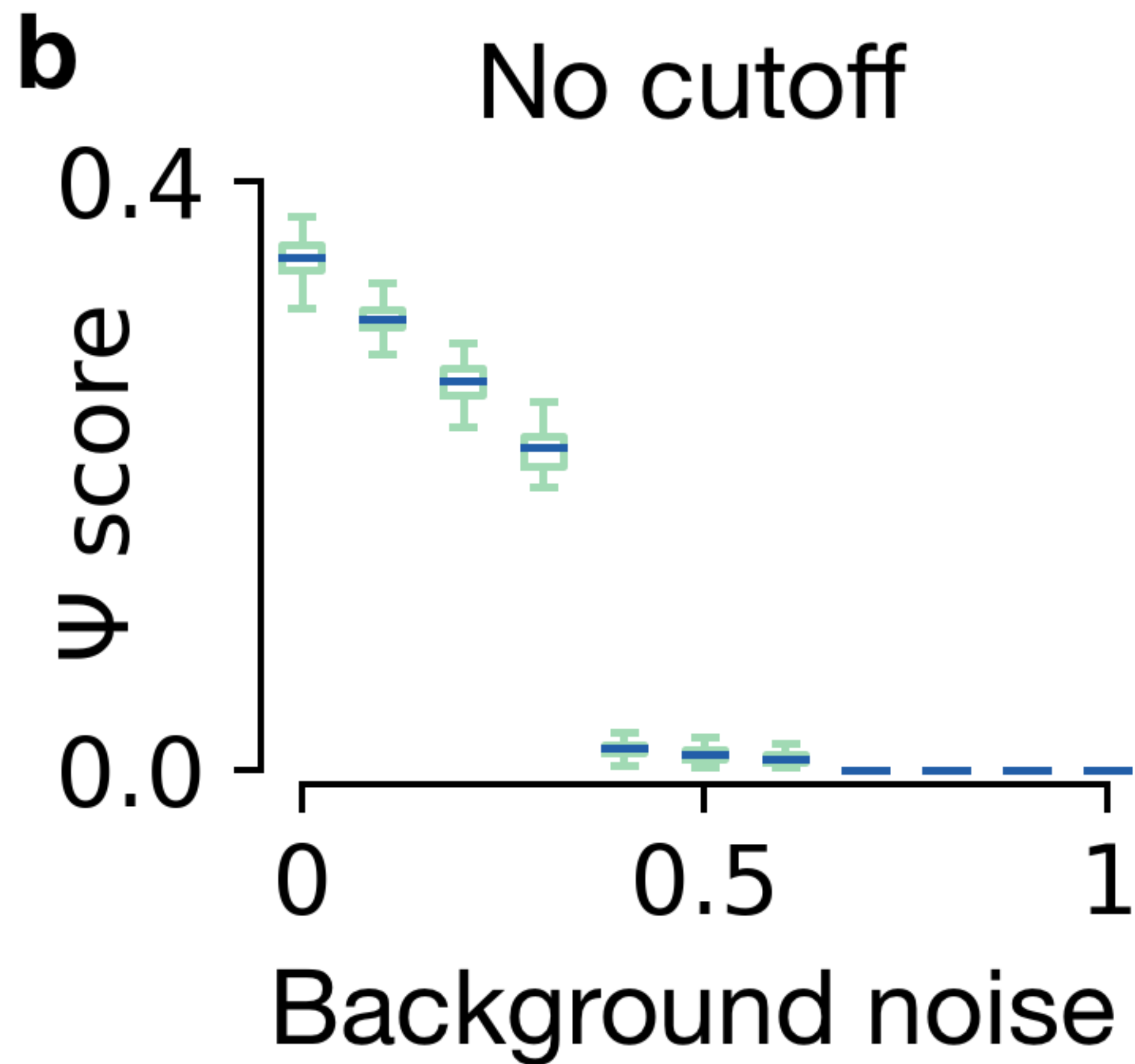

Supplement: S2 Fig — a) Same figure as Fig 4f. Here, the grid spacing for the neighborhood shell is determined by taking the location of the first peak in the pairwise spike distance histogram whose distance value exceeds a cutoff of 15% of the side length of the arena (see Methods for details). b) Same data as in a, but the neighborhood shell is determined using the second peak of the full histogram of pairwise distances between spike locations (without cutting off the first peak). Ψ scores decay to zero at lower noise levels, because the correct detection of the neighborhood shell fails at lower noise levels than in a (see Methods for details). (PDF) [file pcbi.1006804.s002.pdf]
