# Supplementary material for: A local measure of symmetry and orientation for individual spikes of grid cells
Source: PLoS Comput Biol. 2019 Feb 7;15(2):e1006804. doi: 10.1371/journal.pcbi.1006804 (PMC6382163; doi:10.1371/journal.pcbi.1006804)
Supplement: S1 Appendix — Description of the shuffling of spike locations, the generation of spike maps, the computation of the correlogram-based grid score and how we extracted trials with specific light conditions. (PDF) [file pcbi.1006804.s003.pdf]

A LOCAL MEASURE OF SYMMETRY AND ORIENTATION FOR INDIVIDUAL SPIKES OF GRID CELLS

Simon N. Weber[1], Henning Sprekeler[1*],

1 Modelling of Cognitive Processes, Institute of Software Engineering and Theoretical Computer Science, Technische Universität Berlin, Germany

* h.sprekeler@tu-berlin.de

SUPPORTING INFORMATION

Shuffling spike locations for grid cell classification

When classifying grid cells, we use standard shuffling procedures that have been established for conventional correlation-based grid scores (Langston et al., 2010; Wills et al., 2010). To this end, shuffled spike locations are generated from the rat trajectory and the actual spike locations: The times of all spikes are shifted consistently by a temporal offset. This temporal offset is drawn randomly from the interval [20 seconds, time of last spike – 20 seconds] for 100 different shufflings. If, after shuffling, a spike time exceeds the time of the last spike in the original recording, its time is periodically wrapped to the beginning. The shuffled location of each spike is given by the position of the rat at the shifted time of the spike. This shuffling procedure assigns a new location to each spike while maintaining the temporal structure. We classify a cell as a grid cell according to the Ψ score if the Ψ score of the original recording exceeds the 95th percentile of all Ψ scores computed for the 100 shufflings. Correspondingly, to classify a cell as a grid cell according to the ρ score, we use the same procedure but with the ρ score instead of the Ψ score.

Note that the shuffling procedure described above destroys grid fields. Recently, it was suggested to use a different shuffling procedure that corresponds to randomly relocating grid field locations rather than spike locations (Barry and Burgess, 2017)..

Generating artificial spike locations

To generate artificial grid patterns, we draw spikes from a probability distribution that is comprised of two-dimensional Gaussians whose centers lie on a—possibly perturbed—hexagonal grid. The center location of each Gaussian is the location of a grid field. For all generated data, we use an arena size of $1\text{m} \times 1\text{m}$. To comply with the experimental observation that the size of grid fields scales with the grid spacing (Brun et al., 2008), we

use a constant ratio of the width of the Gaussians and the grid spacing before adding perturbations:

$$\text{grid spacing / standard deviation of Gaussian} = 7\,.$$

To assess the robustness of the grid score, we study several perturbations of hexagonal firing patterns. To this end, we first generate grid field locations on a perfect hexagonal grid. To avoid edge effects, this grid is generated on a much larger area that contains a perfect hexagonal arrangement of 271 (i.e., side length 10) grid fields, and the actual arena size ($1\text{m} \times 1\text{m}$) is cut out in the center after the perturbations are applied.

In the scenario of random perturbations (Fig 3a), we add a different random vector to each grid field location. Each such two dimensional vector is drawn from a two dimensional normal distribution. The standard deviation of this distribution dictates the noise on the field locations (Fig 3c).

In the scenario of shearing, we apply a horizontal shearing transformation to each field location of the perfect lattice, by multiplying the two dimensional location of each firing field with the matrix

$$\begin{pmatrix} 1 & s \\ 0 & 1 \end{pmatrix},$$

where s is the strength of the shearing (Fig 4c).

In the scenario of different levels of *background noise*, we create grid patterns with 2000 spikes each. The locations of the 2000 spikes are drawn from two distributions at different fractions. One fraction of spikes is drawn from the above-mentioned perfect hexagonal arrangement of Gaussians. The other fraction is drawn from a spatially homogeneous distribution over the accessible arena. The larger this fraction, the stronger the background noise.

In the scenario of a drift in orientation along the south-north axis, we rotate the location of the i-th grid field $(x_i, y_i)^T$ via

$$\begin{pmatrix} x_i' \\ y_i' \end{pmatrix} = \begin{pmatrix} \cos(\phi_i) & -\sin(\phi_i) \\ \sin(\phi_i) & \cos(\phi_i) \end{pmatrix} \begin{pmatrix} x_i \\ y_i \end{pmatrix},$$

where $(x_i', y_i')^T$ denotes the location of the grid field after rotation and ϕ_i increases along the south-north axis in the following way:

$$\phi_i = d\,(2(y_i - y_{\text{min}})/(y_{\text{max}} - y_{\text{min}}) - 1)\,,$$

where y_{min} and y_{max} are the y-locations of the southernmost and northernmost grid fields, respectively. The factor d determines the strength of the drift. We use $d = \pi/3$, in the example shown in Fig 7c.

Finally, in the scenario of a sudden change in orientation (Fig 7b), we extracted the field locations from simulations by Rosay et al. (2018).

The correlogram-based grid score

To compute the correlogram-based grid score, we use the method suggested by Langston et al. (2010). To this end, we first calculate a "rate map" by summing over Gaussian kernels with a width of 5% of the arena side length, centered at the locations of the spikes.

We then determine the spatial autocorrelogram, i.e., the Pearson correlation coefficients for all spatial shifts of the rate map against itself. From this correlogram, we crop out an annulus that contains the six fields that are arranged around the central peak. To get the inner radius of this annulus, we clip all values in the correlogram that are smaller than 0.1 to 0. We obtain the resulting clusters of values that are larger than 0.1 using `scipy.ndimage.measurements.label` from the *SciPy* package for *Python* with a quadratic filter structure, $((1,1,1),(1,1,1),(1,1,1))$, for a correlogram with 201×201 pixels. We use the distance from the center to the outermost pixel of the innermost cluster as the inner radius of the annulus. We obtain the outer radius of the annulus by trying 50 values, linearly increasing from the inner radius to a corner of the arena. With each of the resulting 50 annuli, we crop the correlogram, rotate it and correlate it with an unrotated copy of the correlogram. We determine the correlation coefficient for 30, 60, 90, 120 and 150 degrees. We define the grid score as the minimum of the correlation values at 60 and 120 degrees minus the maximum of the correlation values at 30, 90 and 150 degrees. After trying all 50 annuli, we take the highest resulting grid score as the grid score of the cell. A hexagonal symmetry thus leads to positive values whereas a quadratic symmetry leads to negative values. Since the correlogram-based score uses Pearson correlation values that are typically expressed with the Greek letter ρ, we refer to it as the ρ score. Note that the match of the grid score symbols with the first names of the authors that introduced them (ρ-samund and Ψ-mon) is coincidental.

Extracting trials with specific light conditions

In the experimental protocol of Pérez-Escobar et al. (2016), different lights on different sides of the arena walls are followed by epochs of darkness. The authors measured different grid patterns when different sides of the wall were illuminated (Pérez-Escobar et al., 2016). In our analysis, we were only interested in the effect of darkness on an individual pattern. We thus concatenated the spikes from trials with one lighting condition with subsequent trials of darkness and did not mix spikes from trials with two different lighting conditions.

Box plot settings

In all shown box plots, each box extends from the first to the third quartile, with a dark blue line at the median. The lower whisker reaches from the lowest data point still within 1.5 IQR of the lower quartile, and the upper whisker reaches to the highest data point still within 1.5 IQR of the upper quartile, where IQR is the inter quartile range between the third and first quartile.

BIBLIOGRAPHY

Barry, C. and Burgess, N. (2017), "To be a Grid Cell: Shuffling procedures for determining 'Gridness'", *bioRxiv*: 230250.

Brun, V. H., Solstad, T., Kjelstrup, K. B., Fyhn, M., Witter, M. P., Moser, E. I., and Moser, M.-B. (2008), "Progressive increase in grid scale from dorsal to ventral medial entorhinal cortex", *Hippocampus*, 18/12: 1200–12.

Langston, R. F., Ainge, J. A., Couey, J. J., Canto, C. B., Bjerknes, T. L., Witter, M. P., Moser, E. I., and Moser, M.-B. (2010), "Development of the spatial representation system in the rat", *Science*, 328/5985: 1576–80.

Pérez-Escobar, J. A., Kornienko, O., Latuske, P., Kohler, L., and Allen, K. (2016), "Visual landmarks sharpen grid cell metric and confer context specificity to neurons of the medial entorhinal cortex", *eLife*, 5: e16937.

Rosay, S., Weber, S., and Mulas, M. (2018), "Modeling grid fields instead of modeling grid cells", *bioRxiv*: 481747.

Wills, T. J., Cacucci, F., Burgess, N., and O'Keefe, J. (2010), "Development of the hippocampal cognitive map in preweanling rats", *Science*, 328/5985: 1573–6.
